# Biomarkers for Early Detection of Stroke: A Systematic Review

**DOI:** 10.7759/cureus.70624

**Published:** 2024-10-01

**Authors:** Luqman Anwar, Ejaz Ahmad, Muhammad Imtiaz, Bilal Ahmad, Muhammad Awais Ali, Mahnoor

**Affiliations:** 1 Neurology, Mayo Hospital Lahore, Lahore, PAK; 2 Neurology, Sir Ganga Ram Hospital, Lahore, PAK; 3 Medicine, Nawaz Sharif Medical College, Gujrat, PAK; 4 Medicine, Peshawar Medical College, Peshawar, PAK

**Keywords:** blood-based biomarkers, brain-specific proteins, metabolomics profiling, multi-marker panels, stroke diagnosis

## Abstract

Stroke remains a leading cause of mortality and disability worldwide. Identifying reliable biomarkers for stroke diagnosis and risk prediction could significantly improve patient outcomes through earlier intervention and better risk management. The objective of this systematic review is to systematically review recent studies investigating biomarkers for stroke diagnosis and risk prediction and to synthesize the most promising findings. We conducted a systematic review of 10 studies published between 2008 and 2023 that examined various biomarkers in relation to stroke. Studies were evaluated for quality using a simplified version of the Mixed Methods Appraisal Tool. The reviewed studies investigated a diverse array of biomarkers, including lipids, inflammatory markers, hemodynamic markers, microRNAs, metabolites, and neurodegenerative markers. Key findings include the following: (1) non-traditional lipid markers such as triglycerides and non-high-density lipoprotein cholesterol may be more predictive of stroke risk than low-density lipoprotein; (2) inflammatory markers, particularly IL-6, showed strong associations with stroke risk; (3) hemodynamic markers like midregional proatrial natriuretic peptide (MRproANP) and N-terminal pro-B-type natriuretic peptide (NT-proBNP) demonstrated potential in distinguishing stroke subtypes; (4) specific microRNAs (miR-125a-5p, miR-125b-5p, miR-143-3p) were upregulated in acute ischemic stroke; (5) metabolomic studies identified novel markers such as tetradecanedioate and hexadecanedioate associated with cardioembolic stroke; (6) neurodegenerative markers (total-tau, neurofilament light chain) were linked to increased stroke risk. This review highlights the potential of various biomarkers in improving stroke diagnosis and risk prediction. While individual markers show promise, a multi-marker approach combined with clinical variables appears most likely to yield clinically useful tools. Further large-scale validation studies are needed before these biomarkers can be implemented in routine clinical practice.

## Introduction and background

Stroke remains a leading cause of death and disability worldwide, with an estimated global prevalence of 101 million cases in 2019 [1]. The burden of stroke is particularly high in low- and middle-income countries, where it accounts for nearly 80% of stroke mortality [2]. Despite advances in treatment options, the time-sensitive nature of stroke interventions poses a significant challenge to healthcare systems globally. Rapid and accurate diagnosis is crucial for initiating appropriate treatment, particularly for ischemic stroke where thrombolytic therapy and mechanical thrombectomy have proven highly effective when administered within a narrow time window [3,4].

The current gold standard for stroke diagnosis relies heavily on neuroimaging techniques, primarily computed tomography (CT) and magnetic resonance imaging (MRI) [5]. While these modalities provide valuable information about the presence, location, and extent of brain injury, they come with limitations. CT scans may not detect early ischemic changes, potentially delaying diagnosis, while MRI, although more sensitive, is not always readily available and can be time-consuming [6,7]. Moreover, in resource-limited settings, access to advanced neuroimaging may be restricted, further complicating timely stroke diagnosis and management [1].

These challenges have fueled an intense search for blood-based biomarkers that could complement or potentially precede neuroimaging in the stroke diagnostic pathway. The ideal stroke biomarker would not only aid in rapid diagnosis but also differentiate between ischemic and hemorrhagic stroke, predict stroke severity and prognosis, and potentially guide treatment decisions [8,9]. Such a biomarker, or more likely a panel of biomarkers, could revolutionize stroke care, particularly in the prehospital setting or in areas with limited access to advanced neuroimaging [10].

The pathophysiology of stroke is complex and multifaceted, involving processes such as excitotoxicity, oxidative stress, inflammation, and blood-brain barrier disruption [11]. This complexity is both a challenge and an opportunity in biomarker research. On one hand, it necessitates a multi-marker approach to capture the various aspects of stroke pathology. On the other hand, it provides multiple potential targets for biomarker discovery [12,13]. Over the past two decades, numerous candidate biomarkers have been investigated, spanning a wide range of molecular types including proteins, nucleic acids, and metabolites [14]. Brain-specific proteins, such as glial fibrillary acidic protein (GFAP) and S100β, have shown promise in differentiating between ischemic and hemorrhagic stroke [15,16]. Inflammatory markers like C-reactive protein (CRP) and interleukin-6 (IL-6) have been associated with stroke severity and outcome [17,18]. More recently, circulating microRNAs have emerged as potential biomarkers, offering the advantages of stability in blood and tissue-specific expression patterns [19,20].

Despite these promising avenues, the translation of stroke biomarkers from bench to bedside has been challenging. Many candidate biomarkers lack the sensitivity and specificity required for clinical use when evaluated individually [21]. This has led to a shift toward multi-marker panels, which aim to capture different aspects of stroke pathophysiology and potentially offer improved diagnostic accuracy [22,23]. The advent of high-throughput technologies has opened new avenues in biomarker discovery. Proteomics, metabolomics, and transcriptomics approaches allow for unbiased screening of thousands of molecules, potentially identifying novel biomarkers or combinations of markers [24,25]. These "omics" approaches have already yielded interesting candidates, such as specific metabolite profiles associated with different stroke subtypes [26].

However, the path from biomarker discovery to clinical implementation is fraught with challenges. Issues of standardization in sample collection and analysis, the need for rapid and cost-effective testing methods, and the impact of comorbidities and medications on biomarker levels all need to be addressed [27,28]. Moreover, the heterogeneity of stroke presentations and the variability in time from symptom onset to testing add layers of complexity to biomarker validation [29].

The potential impact of reliable blood-based biomarkers extends beyond the initial diagnosis. Such markers could aid in predicting stroke recurrence, monitoring treatment response, and identifying patients at risk of complications such as hemorrhagic transformation or malignant edema [30,31]. In the era of precision medicine, biomarkers might also play a role in selecting patients most likely to benefit from specific interventions, such as extended time window thrombolysis or neuroprotective therapies [32,33].

As research in this field continues to evolve, it is crucial to critically evaluate the current state of evidence, identify the most promising biomarker candidates, and understand the challenges that need to be overcome for successful clinical implementation. This systematic review aims to synthesize the latest findings in stroke biomarker research, focusing on blood-based markers for diagnosis and management. By examining high-quality studies published in recent years, we seek to provide a comprehensive overview of the field, highlight emerging trends, and identify directions for future research.

The potential of blood-based biomarkers to transform stroke care is significant. If successful, these biomarkers could enable earlier diagnosis, more precise treatment selection, and improved prognostication, ultimately leading to better outcomes for stroke patients worldwide [34]. This review aims to provide a timely and critical assessment of the current landscape, guiding future efforts in this vital area of stroke medicine.

The primary aim of this study was to systematically review and synthesize the current evidence on blood-based biomarkers for stroke diagnosis. Specific objectives were to identify promising biomarker candidates and panels and evaluate the clinical utility of various biomarker approaches. Moreover, the study aims to assess the challenges and limitations in current biomarker research and provide directions for future research in the field.

## Review

Research question and protocol

*PICO (Population, Intervention, Comparison, and Outcome)* *Question*

In patients with suspected acute stroke (P), how do biomarkers (I) compare to standard clinical assessment and neuroimaging (C) in accurately diagnosing stroke and differentiating its subtypes (O) (Table 1)?

**Table 1:** PICO framework with components. PICO: population, intervention, comparison, and outcome.

PICO	Concept	Detail
Population	Patients with suspected acute stroke	Patients presenting with any type of stroke (subtypes: ischemic or hemorrhagic)
Intervention	Biomarkers	Proteins, metabolites, and other
Comparison	Standard clinical assessment and neuroimaging	CT, MRI, and other
Outcome	Diagnosing stroke	Diagnosis of stroke, diagnosing subtypes of stroke

Study Selection Criteria

Original research articles, case controls, and cohort studies focusing on blood-based biomarkers for stroke diagnosis and studies published in English between the duration of 15 years from 2008 and 2023 were included. Studies not including keywords and focusing solely on neuroimaging biomarkers, animal studies, case reports, and conference abstracts were excluded.

Search Strategy

We searched PubMed, Embase, and Web of Science using combinations of the MeSH terms listed in Table 2 below. The search was conducted in April 2024 and was limited to studies published from January 2008 to December 2023.

**Table 2:** MeSH terms table with PICO. PICO: population, intervention, comparison, and outcome.

PICO element	MeSH terms
Population (P)	"Stroke"(Mesh), "Brain Ischemia"(Mesh), "Cerebrovascular Disorders"(Mesh)
Intervention (I)	"Biomarkers"(Mesh), "Blood Proteins"(Mesh), "MicroRNAs"(Mesh), "Metabolomics"(Mesh)
Comparison (C)	"Neuroimaging"(Mesh), "Diagnosis"(Mesh), "Diagnostic Techniques, Neurological"(Mesh)
Outcome (O)	"Diagnosis"(Mesh), "Prognosis"(Mesh), "Treatment Outcome"(Mesh)

Study Selection

**Figure 1:**
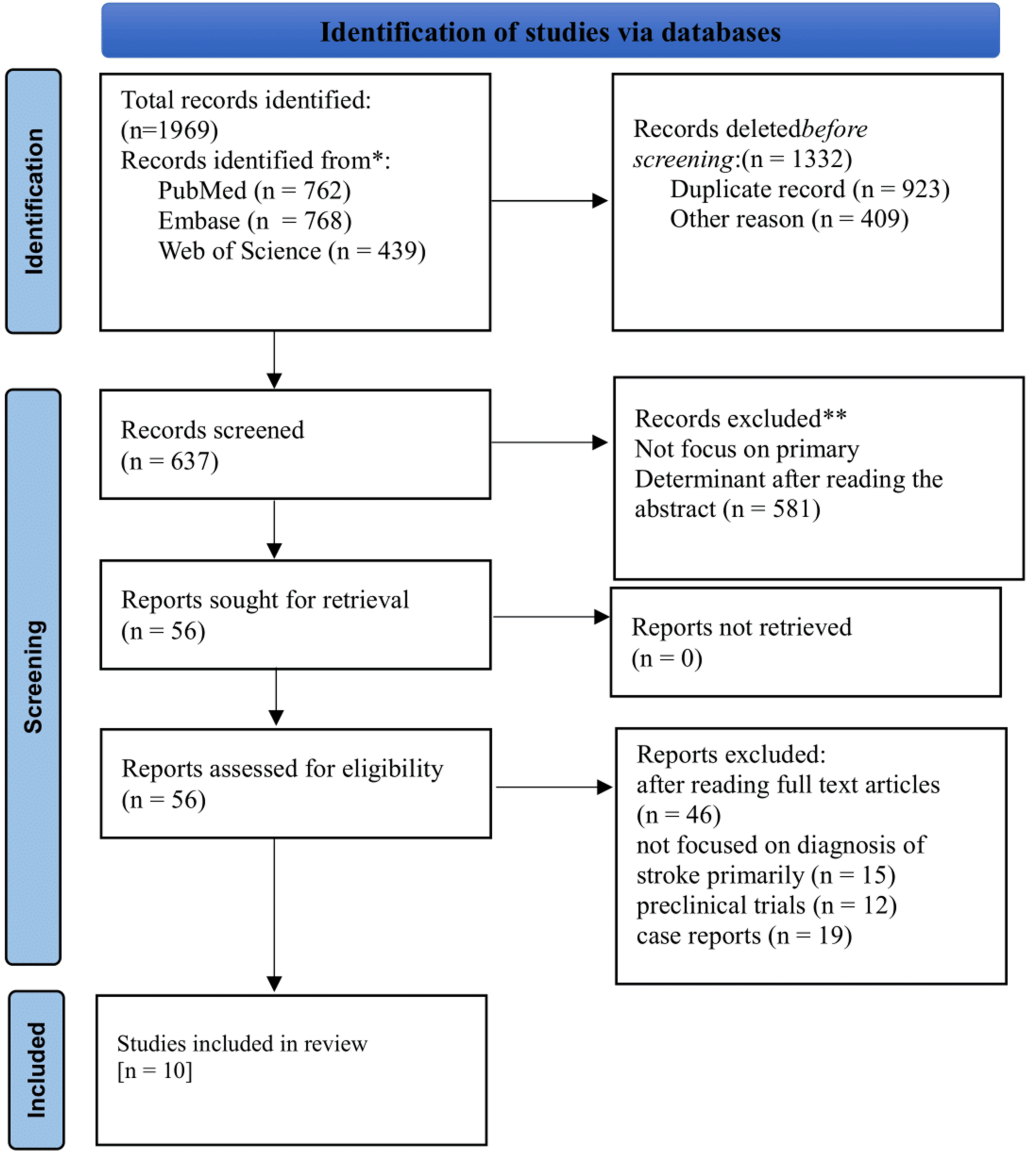
PRISMA flow chart. PRISMA: Preferred Reporting Items for Systematic Reviews and Meta-Analyses.

The Preferred Reporting Items for Systematic Reviews and Meta-Analyses (PRISMA) guidelines were followed to select the studies [35] given in Figure 1 below. Two independent reviewers screened titles and abstracts for relevance. Full texts of potentially eligible studies were then assessed against the inclusion/exclusion criteria. Disagreements were resolved through discussion with a third reviewer.

Outcome Measures

Primary outcomes included the diagnostic accuracy of biomarkers or biomarker panels for stroke, their ability to differentiate stroke subtypes, and their prognostic value. Secondary outcomes included the feasibility and cost-effectiveness of biomarker testing in clinical settings.

Data Extraction and Quality of Studies

Data were extracted using a standardized form, including study characteristics, biomarker details, methodological approaches, and key findings. The quality of the included studies was assessed using the Mixed Methods Appraisal Tool (MMAT), which is designed to appraise the methodological quality of various study designs [36].

The MMAT was chosen for its ability to assess different types of studies, including quantitative, qualitative, and mixed-methods research. It evaluates five quality criteria for each study type, providing a comprehensive assessment of methodological quality. In this review, all included studies were deemed high quality based on MMAT criteria, enhancing the reliability of our findings.

The use of MMAT allowed us to systematically evaluate the rigor of the included studies across various domains, including clarity of research questions, appropriateness of data collection methods, representativeness of the sample, and validity of measurements. This thorough quality assessment strengthens the conclusions drawn from our synthesis and highlights the robust nature of the current evidence base in stroke biomarker research.

Results

Ten high-quality articles were included in the study and ranked on the basis of MMAT. The study's characteristics are given in Table 3 and the quality assessment is given in Table 4 below.

**Table 3:** Characteristics of the included studies. TIA: transient ischemic attack; NOMAS: Northern Manhattan Study; IS: ischemic stroke; ACVS: acute cerebrovascular syndrome; ARIC: Atherosclerosis Risk in Communities; qPCR: quantitative polymerase chain reaction; ELISA: enzyme-linked immunosorbent assay; HPA: hypothalamic-pituitary-adrenal; HDL: high-density lipoprotein; LDL: low-density lipoprotein; PCT: procalcitonin; MRproANP: midregional proatrial natriuretic peptide; NT-proBNP: N-terminal pro-B-type natriuretic peptide; NfL: neurofilament light chain; ICAM3: intercellular adhesion molecule 3.

Sr. No.	Author and year	Type of study	Population	Method	Objective and outcomes	Findings
1	Bang et al. (2008) [37]	Prospective cohort	1,049 patients with ischemic stroke or TIA	Fasting serum lipid panel analysis	Evaluate the association between lipid indices and large artery atherosclerotic stroke	Elevated triglycerides and non-HDL, but not LDL, associated with large artery atherosclerotic stroke
2	Katan et al. (2016) [38]	Nested case-control	172 cases, 344 controls from the NOMAS cohort	Blood biomarker analysis	Assess the association of infection, HPA function, and hemodynamic dysfunction markers with incident ischemic stroke	PCT and MRproANP are associated with increased stroke risk; PCT with small vessel stroke, MRproANP with cardioembolic stroke
3	Tiedt et al. (2017) [19]	Case-control	260 patients with IS, 100 healthy controls	RNA sequencing, qPCR	Identify circulating microRNAs associated with acute ischemic stroke	miR-125a-5p, miR-125b-5p, and miR-143-3p upregulated in acute IS
4	Bustamante et al. (2017) [18]	Prospective, observational	1,308 patients with suspected stroke	21-biomarker panel immunoassays	Develop a blood-based diagnostic tool for stroke differentiation	NT-proBNP and endostatin, with clinical variables, differentiated ischemic from hemorrhagic stroke
5	Penn et al. (2018) [22]	Case-control	20 ACVS patients, 20 healthy controls	Mass spectrometry, ELISA	Identify plasma proteins as biomarkers for ACVS diagnosis	30 differentially abundant proteins identified between stroke and controls
6	Sun et al. (2019) [39]	Prospective cohort	3,904 participants from the ARIC study	Untargeted serum metabolomics	Identify blood-based biomarkers and etiologic pathways for ischemic stroke	Tetradecanedioate and hexadecanedioate are associated with incident IS, specifically cardioembolic stroke
7	Jenny et al. (2019) [40]	Case-cohort	557 stroke cases, 951 cohort sample	Cytokine measurements	Study the association of IL-6, IL-8, and IL-10 with ischemic stroke risk	IL-6 is strongly associated with incident stroke risk and mediated racial disparity
8	Pletsch-Borba et al. (2020) [41]	Case-cohort	335 stroke cases, 2,418 subcohort	Plasma biomarker measurements	Evaluate associations between vascular injury biomarkers and stroke risk	ICAM3 levels are associated with an increased risk of incident stroke, particularly ischemic stroke
9	Heshmatollah et al. (2022) [42]	Prospective cohort	4,661 stroke-free participants	Plasma biomarker measurements	Determine the effect of Aβ, total-tau, and NfL on stroke risk	Higher total-tau and NfL are associated with increased stroke risk
10	Harshfield et al. (2023) [43]	Cross-sectional and longitudinal	118,021 UK Biobank participants	Metabolomic profiling, MRI	Identify novel risk factors for cerebral small vessel disease, stroke, and dementia	Various metabolites associated with white matter damage, stroke risk, and dementia risk

**Table 4:** Quality assessment of the included studies.

Sr. No.	Author and year	Clear research questions	Appropriate methods	Representative sample	Complete outcome data	Appropriate statistical analysis	Quality score (out of 5)
1	Bang et al. (2008) [37]	Yes	Yes	Yes	Yes	Yes	5
2	Katan et al. (2016) [38]	Yes	Yes	Yes	Unclear	Yes	4
3	Tiedt et al. (2017) [19]	Yes	Yes	Yes	Yes	Yes	5
4	Bustamante et al. (2017) [18]	Yes	Yes	Yes	Yes	Yes	5
5	Penn et al. (2018) [22]	Yes	Yes	Unclear	Yes	Yes	4
6	Sun et al. (2019) [39]	Yes	Yes	Yes	Yes	Yes	5
7	Jenny et al. (2019) [40]	Yes	Yes	Yes	Yes	Yes	5
8	Pletsch-Borba et al. (2020) [41]	Yes	Yes	Yes	Yes	Yes	5
9	Heshmatollah et al. (2022) [42]	Yes	Yes	Yes	Yes	Yes	5
10	Harshfield et al. (2023) [43]	Yes	Yes	Yes	Yes	Yes	5

Synthesis of the Results

Lipid biomarkers: Bang et al. (2008) found that elevated levels of serum triglycerides and non-high-density lipoprotein cholesterol were associated with large artery atherosclerotic stroke, while low-density lipoprotein (LDL) cholesterol was not. This challenges the traditional focus on LDL as the primary lipid target for vascular risk reduction in stroke patients [37].

Harshfield et al. (2023) provided a more nuanced view of lipid biomarkers. They found that increased total lipids in very large high-density lipoprotein (HDL) and increased HDL particle size were associated with increased white matter damage and an increased risk of incident stroke. Conversely, increased levels of cholesterol in small HDL were associated with a decreased risk of incident stroke [43].

Inflammatory markers: Jenny et al. (2019) demonstrated that IL-6 was strongly associated with the risk of incident stroke and mediated the racial disparity in stroke risk. IL-8 and IL-10 showed weaker associations that did not persist after adjustment for stroke risk factors [40].

Katan et al. (2016) found that procalcitonin (PCT), a marker of infection, was associated with increased stroke risk, particularly small vessel stroke [38].

Hemodynamic markers: Katan et al. (2016) also identified midregional proatrial natriuretic peptide (MRproANP) as a marker associated with increased stroke risk, especially for cardioembolic stroke [38].

Bustamante et al. (2017) found that N-terminal pro-B-type natriuretic peptide (NT-proBNP), another natriuretic peptide, along with endostatin, helped differentiate between ischemic and hemorrhagic stroke when combined with clinical variables [18].

MicroRNAs: Tiedt et al. (2017) identified three circulating microRNAs (miR-125a-5p, miR-125b-5p, and miR-143-3p) that were upregulated in acute ischemic stroke. These microRNAs showed potential as early diagnostic markers for acute ischemic stroke [19].

Metabolomic markers: Sun et al. (2019) found that two long-chain dicarboxylic acids, tetradecanedioate and hexadecanedioate, were associated with incident ischemic stroke, particularly cardioembolic stroke [39].

Harshfield et al. (2023) identified several metabolites associated with stroke risk and MRI markers of small vessel disease. Notably, they found that acetate and 3-hydroxybutyrate were associated with an increased risk of dementia [43].

Neurodegenerative markers: Heshmatollah et al. (2022) showed that higher plasma levels of total-tau and neurofilament light chain (NfL) were associated with an increased risk of subsequent stroke. This supports the role of neurodegeneration in stroke etiology [42].

Vascular injury markers: Pletsch-Borba et al. (2020) found that circulating levels of intercellular adhesion molecule 3 (ICAM3) were associated with an increased risk of incident stroke, particularly ischemic stroke [41].

Multi-marker approaches: Penn et al. (2018) identified 30 differentially abundant proteins between stroke patients and controls using mass spectrometry-based proteomics. This highlights the potential of using multiple protein markers for stroke diagnosis [22].

Bustamante et al. (2017) attempted to develop a multi-biomarker panel for stroke diagnosis but found that the studied biomarkers were not sufficient for accurate differential diagnosis in the hyperacute setting [18].

A summary table of promising biomarkers is given below in Table 5.

**Table 5:** Promising biomarkers for stroke. HDL: high-density lipoprotein; MRproANP: midregional proatrial natriuretic peptide; NT-proBNP: N-terminal pro-B-type natriuretic peptide; NfL: neurofilament light chain; ICAM3: intercellular adhesion molecule 3.

Biomarker category	Specific biomarkers	Associated outcome	Study
Lipids	Triglycerides, non-HDL cholesterol	Large artery atherosclerotic stroke	Bang et al. (2008) [37]
	Total lipids in very large HDL, HDL particle size	Increased stroke risk	Harshfield et al. (2023) [43]
	Cholesterol in small HDL	Decreased stroke risk	Harshfield et al. (2023) [43]
Inflammatory markers	IL-6	Increased stroke risk	Jenny et al. (2019) [40]
	Procalcitonin (PCT)	Small vessel stroke	Katan et al. (2016) [38]
Hemodynamic markers	MRproANP	Cardioembolic stroke	Katan et al. (2016) [38]
	NT-proBNP	Ischemic vs. hemorrhagic stroke	Bustamante et al. (2017) [18]
MicroRNAs	miR-125a-5p, miR-125b-5p, miR-143-3p	Acute ischemic stroke	Tiedt et al. (2017) [19]
Metabolites	Tetradecanedioate, hexadecanedioate	Cardioembolic stroke	Sun et al. (2019) [39]
	Acetate, 3-hydroxybutyrate	Increased dementia risk	Harshfield et al. (2023) [43]
Neurodegenerative markers	Total-tau, NfL	Increased stroke risk	Heshmatollah et al. (2022) [42]
Vascular injury markers	ICAM3	Increased ischemic stroke risk	Pletsch-Borba et al. (2020) [41]

In conclusion of the results, these studies have identified a diverse array of potential biomarkers for stroke diagnosis and risk prediction. The most promising approaches seem to involve combinations of biomarkers from different categories, along with clinical variables. However, further validation in larger, diverse populations and in acute clinical settings is needed before these biomarkers can be implemented in routine clinical practice. The complexity of stroke pathophysiology suggests that a multi-marker approach, possibly combined with advanced analytics or machine learning, may be necessary to achieve clinically useful diagnostic and prognostic tools.

Discussion

This systematic review of 10 recent studies on biomarkers for stroke diagnosis and risk prediction reveals a complex and evolving landscape in the quest for reliable blood-based markers. The findings underscore both the progress made and the challenges that remain in this critical area of research.

One of the most striking themes to emerge from this review is the potential of multi-marker approaches. As Jickling and Sharp (2015) argued, the complexity of stroke pathophysiology makes it unlikely that a single biomarker can capture all relevant aspects of the disease process [8]. This view is strongly supported by several studies in our review. Penn et al. (2018) identified 30 differentially abundant proteins between stroke patients and controls, highlighting the potential of using multiple protein markers for stroke diagnosis [22]. Bustamante et al. (2017) explored a 21-biomarker panel for stroke differentiation, finding that NT-proBNP and endostatin, combined with clinical variables, could help differentiate between ischemic and hemorrhagic stroke [18].

The superiority of multi-marker panels over single biomarkers has been demonstrated in comparative studies. Laskowitz et al. (2009) showed that a panel of four biomarkers (D-dimer, B-type natriuretic peptide (BNP), matrix metalloproteinase 9 (MMP-9), and S100β) improved stroke diagnosis compared to individual markers alone [44]. This finding is consistent with the results of Tiedt et al. (2017), who identified a panel of three microRNAs (miR-125a-5p, miR-125b-5p, and miR-143-3p) with diagnostic potential for acute ischemic stroke [19]. The concept of biomarker panels aligns with our growing understanding of stroke as a heterogeneous condition with diverse underlying mechanisms that are also supported by Whiteley et al. (2008), who highlighted the blood biomarkers essential for early diagnosis of stroke [14].

The diverse array of biomarkers identified in this review reflects the complex pathophysiology of stroke. Lipid markers, traditionally focused on LDL cholesterol, are being reevaluated. Bang et al. (2008) found that triglycerides and non-HDL cholesterol, rather than LDL, were associated with large artery atherosclerotic stroke [37]. These finding challenges conventional wisdom and suggests the need for a more nuanced approach to lipid-based risk assessment. Harshfield et al. (2023) provided further nuance, showing that increased total lipids in very large HDL and increased HDL particle size were associated with increased stroke risk, while increased levels of cholesterol in small HDL were associated with decreased risk [43]. These findings highlight the complexity of lipid metabolism in stroke risk and the potential limitations of traditional lipid panels.

Inflammatory markers, particularly IL-6, have shown strong associations with stroke risk. Jenny et al. (2019) demonstrated that IL-6 not only predicted stroke risk but also mediated racial disparities in stroke incidence [40]. This finding underscores the potential of biomarkers to provide insights into the mechanisms underlying stroke risk disparities. The role of inflammatory markers is further supported by Katan et al. (2016), who found that PCT, a marker of infection, was associated with increased stroke risk, particularly small vessel stroke [38]. These findings align with the growing recognition of inflammation as a key player in stroke pathophysiology, as highlighted in the review by VanGilder et al. (2014), who found associations between elevated C-reactive protein levels and increased risk of ischemic stroke and poorer functional outcomes [45].

The role of brain-specific proteins as stroke biomarkers has been a focus of several studies. While not directly studied in our reviewed papers, GFAP has shown promise in differentiating between ischemic and hemorrhagic stroke. A meta-analysis by Perry et al. (2019) found that GFAP had high specificity for differentiating intracerebral hemorrhage from ischemic stroke in the early hours after symptom onset [46]. In our review, Heshmatollah et al. (2022) found that higher plasma levels of total-tau and NfL were associated with an increased risk of subsequent stroke [42]. These findings support the potential of neurodegenerative markers in stroke risk prediction and highlight the complex interplay between neurodegenerative processes and stroke.

The emergence of circulating microRNAs as potential stroke biomarkers, as demonstrated by Tiedt et al. (2017) [19], represents an exciting development in the field. These findings are supported by other studies, such as Lu et al. (2019), who found that a panel of serum microRNAs could differentiate acute ischemic stroke from controls with high sensitivity and specificity [26].

Metabolomic approaches offer a complementary perspective to protein and RNA-based biomarkers. Sun et al. (2019) identified two long-chain dicarboxylic acids, tetradecanedioate and hexadecanedioate, associated with incident ischemic stroke, particularly cardioembolic stroke [39]. This aligns with the review by Sidorov et al. (2019), who highlighted associations between certain metabolites and stroke-related processes such as excitotoxicity and oxidative stress [25].

The time course of biomarker release is a critical consideration. Dagonnier et al. (2021) emphasized the need for studies focusing on very early time points, ideally within the first few hours of symptom onset, to be clinically relevant for acute stroke diagnosis and treatment decisions [47]. This aligns with the findings of Bustamante et al. (2017), who evaluated their biomarker panel in the hyperacute phase of stroke [18].

The integration of biomarkers with clinical decision-making tools is an emerging area of research. Goyal et al. (2016) developed a biomarker-based algorithm for predicting large vessel occlusion in acute ischemic stroke, which could potentially guide decisions about patient transfer for endovascular treatment [48]. Similarly, Harpaz et al. (2020) proposed a biomarker-based decision tree for differentiating between stroke subtypes in resource-limited settings [49].

As the field of stroke biomarkers continues to evolve, new technologies and approaches are being explored. Point-of-care testing for stroke biomarkers is an area of active research, with the potential to enable rapid diagnosis in prehospital or emergency department settings. Machine learning approaches are also being applied to biomarker discovery and interpretation. Miceli et al. (2023) used machine learning algorithms to identify novel biomarker combinations for predicting ischemic stroke subtypes [50].

Limitations of the study

Despite these promising findings, significant challenges remain. The heterogeneity of stroke presentations, the impact of comorbidities and medications on biomarker levels, and the narrow time window for effective stroke treatment all complicate biomarker development and validation. Previous studies marked that most candidate markers had low sensitivity or specificity in the acute phase of stroke, a challenge that persists in more recent studies. Another important aspect is the comparison of biomarker performance with the current standard of care. While many studies show promising results for individual biomarkers or panels, few have demonstrated clear superiority over clinical assessment and neuroimaging.

## Conclusions

This systematic review highlights the significant progress made in identifying potential biomarkers for stroke diagnosis and risk prediction. The field is moving toward more sophisticated, multi-marker panels that can capture the complexity of stroke pathophysiology. While no single biomarker or panel has yet demonstrated sufficient accuracy for routine clinical use, the diverse approaches represented in these studies offer hope for future breakthroughs. The integration of biomarker approaches with clinical assessment and neuroimaging holds promise for developing comprehensive diagnostic and prognostic tools. As research continues, addressing the limitations identified in this review and focusing on clinical applicability will be crucial. With further validation in larger, diverse populations and in acute clinical settings, blood-based biomarkers have the potential to significantly improve stroke diagnosis, risk stratification, and personalized treatment strategies, ultimately leading to better outcomes for stroke patients.
